# The Pseudomonas Quinolone Signal (PQS) Balances Life and Death in *Pseudomonas aeruginosa* Populations

**DOI:** 10.1371/journal.ppat.1000166

**Published:** 2008-09-26

**Authors:** Susanne Häussler, Tanja Becker

**Affiliations:** Department of Cell Biology, Helmholtz Center for Infection Research, Braunschweig, Germany; Massachusetts General Hospital, United States of America

## Abstract

When environmental conditions deteriorate and become inhospitable, generic survival strategies for populations of bacteria may be to enter a dormant state that slows down metabolism, to develop a general tolerance to hostile parameters that characterize the habitat, and to impose a regime to eliminate damaged members. Here, we provide evidence that the pseudomonas quinolone signal (PQS) mediates induction of all of these phenotypes. For individual cells, PQS, an interbacterial signaling molecule of *Pseudomonas aeruginosa*, has both deleterious and beneficial activities: on the one hand, it acts as a pro-oxidant and sensitizes the bacteria towards oxidative and other stresses and, on the other, it efficiently induces a protective anti-oxidative stress response. We propose that this dual function fragments populations into less and more stress tolerant members which respond differentially to developing stresses in deteriorating habitats. This suggests that a little poison may be generically beneficial to populations, in promoting survival of the fittest, and in contributing to bacterial multi-cellular behavior. It further identifies PQS as an essential mediator of the shaping of the population structure of *Pseudomonas* and of its response to and survival in hostile environmental conditions.

## Author Summary

A little poison may be generically beneficial to bacterial populations. By eliminating damaged members and by promoting survival of the fittest, selective poisoning may significantly contribute to multi-cellular bacterial behavior. Here, we report that the pseudomonas quinolone signal (PQS) exhibits both beneficial and deleterious activities and propose that pro- and anti-oxidant effects fragment the population into less and more stress tolerant members. We suggest our findings point to PQS as a cellular “trainer” whose role is to mediate selection of the fittest through a “make or break” mechanism. PQS thereby issues decisions on life and death in *Pseudomonas aeruginosa* populations, and may be essential in shaping the population structure, contributing to multi-cellular development processes in bacterial biofilms, and helping to maintain discrete, ordered spatial structures.

## Introduction

Infectious diseases are the result of a dynamic interplay between the pathogen and the host. The host responds to an infection by activating diverse innate and adaptive immune defence mechanisms, whereas the disease causing pathogen produces an arsenal of virulence factors and evades the activated immune system by a great variety of adaptive responses. Today, the treatment of infectious diseases poses not only problems due to multiresistance but also problems due to the emergence of persisting bacteria that survive within biofilm structures in the human host [1].

Free-floating bacteria usually adapt to distinct environmental conditions with a characteristic change in their gene expression pattern. Different stresses may induce diverse but often overlapping stress responses. However, although many of adaptation strategies operate at the individual cell level, others operate at the population level. In the last decade, proof of bacterial interactiveness has accumulated from the observation of complex multicellular development phenomena in fruiting bodies, sporulation and biofilm formation [2]–[6]. Living in populations provides a species with additional mechanisms of adaptation. Collectively and coordinately, bacteria act far more efficiently than they could as single cells. Thus, multicellularity was proposed as a general bacterial trait that has been increasingly witnessed and that affects multiple phenotypes. The social multicellular behavior in prokaryotes is supported by the identification of signaling molecules that mediate cooperative traits and coordinated behavior [7]. A coordinated expression of genes involved in virulence and persistence is crucial in host-pathogen interactions. During chronic infections many bacteria adopt a biofilm mode of growth with bacteria embedded in an exopolymeric matrix. This mode of growth acts as a protective niche and helps the bacteria to evade the host immune response and to withstand antimicrobial therapy [8]. In *Pseudomonas aeruginosa*, biofilm formation and the production of various virulence determinants are regulated via the action of a hierarchical quorum-sensing system mediated by the two chemically distinct classes of signal molecules, the *N*-acylhomoserine lactones [9] and the 4-quinolones [10]–[14]. The latter group consists of more than 50 compounds [15] and includes the most active signal molecule 2-heptyl-3-hydroxy-4-quinolone which is commonly referred to as the pseudomonas quinolone signal (PQS) [14]. PQS regulates its own production by controlling the expression of the 4-quinolone biosynthetic genes [16] and its own package into membrane vesicles that deliver antimicrobials and toxins [17], transport DNA and might be involved in enhanced survival upon challenge with stressing agents [18].

Stress responses involve considerable investment of cellular resources, often under conditions when cellular resources are limiting and diminishing, a process that may be unsustainable. Thus, a mechanism of uneven distribution of resources in the population, to the benefit of the fittest, may become inevitable. Phenotypically, this would mean that perception of the same stress by the different cells of a population would have very different outcomes, either cell damage, or an increase in tolerance of the stress and an increased fitness and probability of survival. In this study we provide evidence that, although PQS sensitizes the bacteria towards exogenous stresses, PQS also catalyses an efficient bacterial anti-stress response. Our findings add a new dimension to bacterial multicellular behaviour and identifies PQS as an essential factor to shape the population structure and to aid bacterial population adaptation to conditions of increased stress that may significantly contribute to bacterial persistence during chronic infectious diseases.

## Results

### The pseudomonas quinolone signal (PQS) increases bacterial sensitivity to stressful conditions

Considering the finding that autolysis in aging *P. aeruginosa* colonies has been observed to correlate with the level of PQS produced by the bacteria [19], we hypothesized that PQS may act as an endogenous stress factor, and that this endogenous stressor may be deleterious above a threshold level (such as in PQS over-producing colonies that develop an autolytic phenotype) or if the cells encounter additional exogenous stresses.

**Figure 1:**
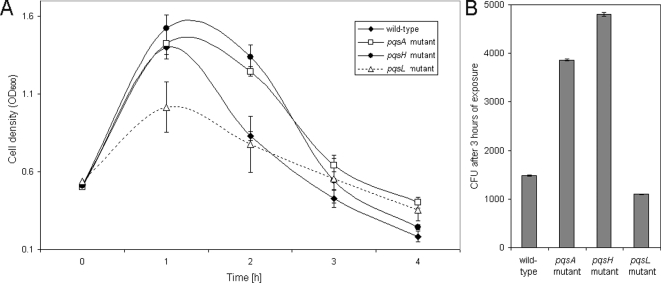
Antibiotic tolerance in the absence of PQS production. Killing curves of log phase grown *P. aeruginosa* PAO1 cultures treated with 5 µg/ml ciprofloxacin were recorded. Killing was significantly delayed in the *pqsA* and *pqsH* mutant compared to the wild-type and the *pqsL* mutant as determined by OD_600_ determinations (A) and CFU counts after 3 h of exposure (B). Error bars mark the standard deviation of three independent experiments. CFU counts of the PAO1 wild-type and the *pqsL* mutant were significantly different (p<0.05, as determined by t-test) as compared to the *pqsA* and *pqsH* mutants.

In order to further elucidate PQS specific effects and to differentiate them from the effects of other 4-quinolones, we measured the sensitivity of wild-type *P. aeruginosa* PAO1, an isogenic 4-quinolone non-producing *pqsA* mutant, a 4-quinolone positive but PQS negative *pqsH* mutant [20], and a PQS overproducing *pqsL* mutant [19] towards the activity of ciprofloxacin and hydrogen peroxide. Whereas the PAO1 wild-type exhibited a steep fall in viability, measured as a decrease in optical density (Figure 1A) and colony forming units (Figure 1B) following exposure to ciprofloxacin, the *pqsA* and the *pqsH* mutant exhibited measurably increased tolerance as the fall in optical density and surviving bacteria exhibited a significant delay. The *pqsL* mutant was found to be most sensitive against the activity of ciproflocxacin. Growth of the wild-type, the mutants and the complemented strain without the addition of the antibiotic was shown to be comparable (Figure S1). Complementation of the *pqsA* mutant, through provision of the *pqsA-E* operon *in trans* (on plasmid pLG10 [20], kindly provided by Colin Manoil), eliminated this tolerance (Figure S2A). The *pqsA* mutant strain also exhibited an enhanced antibiotic tolerance towards imipenem and gentamicin (Figures S2B and C).

**Figure 2:**
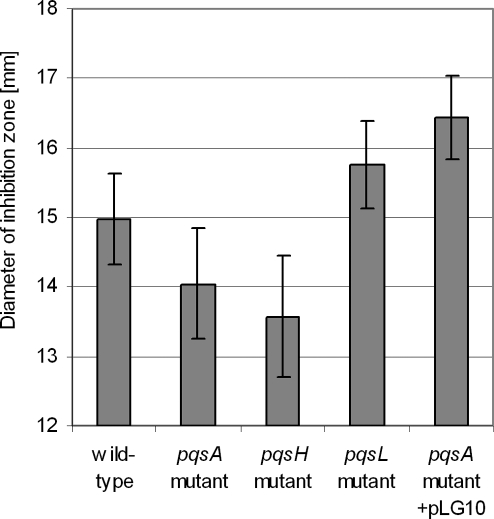
Growth inhibition by H_2_O_2_ as determined by agar diffusion assays. PQS non-producing strains (*pqsA* and *pqsH* mutant) exhibited an increased resistance towards H_2_O_2_ as compared to the wild-type (paired t-test; p-value <0.005). The diameter of the inhibition zone is given as the mean+SD of a triplicate.

We have previously shown that the addition of exogenous PQS enhanced the susceptibility of *P. aeruginosa* towards the bactericidal activity of hydrogen peroxide [21]. In agreement with this observation, we demonstrate here that both the *pqsH* and the *pqsA* mutant exhibited an increased resistance towards the activity of hydrogen peroxide as compared to the PAO1 wild-type, whereas the complemented *pqsA* mutant as well as the PQS overproducing *pqsL* mutant exhibited an increased sensitivity (Figure 2). These results clearly demonstrate that the 4-quinolone PQS sensitizes the bacteria towards the bactericidal activity of antibiotics and oxidative stress.

If the mediation of an endogenous stress that predisposes the bacteria to developing exogenous stresses is a significant contribution of endogenously-produced PQS, what is the situation for exogenously produced PQS, since a key role of PQS is thought to be interbacterial signaling?

**Figure 3:**
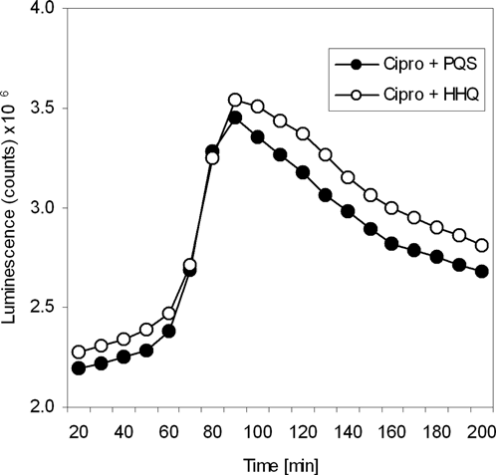
Loss of bioluminescence of *P. aeruginosa* PAO1 wild-type after exposure to the antibiotic ciprofloxacin. Killing of the bacteria by 5 µg/ml ciprofloxacin was significantly enhanced under the addition of PQS as opposed to HHQ (paired t-test; p value <0.05).

To test this, we added synthetic PQS and the biologically less active PQS precursor 4-quinolone 2-heptyl-4-hydroxy-quinoline (HHQ) to the PAO1 wild-type harboring a *luxCDABE* operon *in trans*, and measured the decrease in bioluminescence after exposure to the antibiotic ciprofloxacin. In contrast to the addition of HHQ, killing by cirpofloxacin was significantly enhanced by the simultaneous addition of PQS (Figure 3). These results implicate that PQS but not HHQ increases bacterial sensitivity to exogenous stresses.

### PQS synchronizes the entry of the whole bacterial population into stationary phase

**Figure 4:**
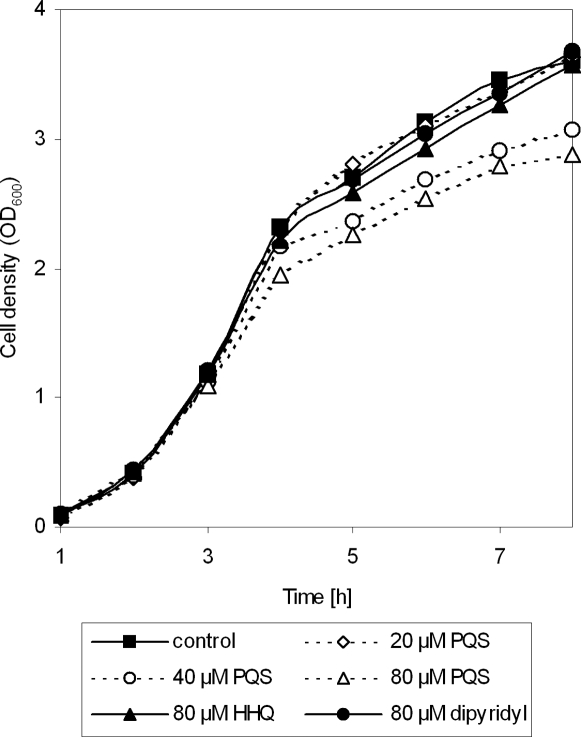
PQS induces bacteriostasis in a concentration dependent manner. The addition of PQS to bacterial cultures of a *pqsA* mutant is characterized by a slope reduction in log phase of growth and lower maximum OD_600_ values as compared to the cultures grown without added PQS. Bacteriostasis seems to be a PQS-specific effect because both the addition of equimolar concentrations of the biologically less active PQS precursor heptyl-4-hydroxy-quinoline (HHQ) or the iron chelator dipyridyl did not have the same effect. This is a representative experiment for at least three independent measurements.

We furthermore noticed that PQS affects bacterial growth. To rule out that PQS induced the production of extracellular factors that might impede on bacterial growth, we added increasing concentrations of PQS to exponentially-growing cultures of a 4-quinolone non-producing and non-responding *pqsA* PAO1 transposon mutant. As depicted in Figure 4, the addition of increasing PQS concentrations led to decreasing growth rates and decreasing maximum OD_600_ values (Figure 4 and [11]), whereas the addition of equimolar concentrations of HHQ or the iron chelator dipyridyl (PQS has been demonstrated to be an iron chelator [21],[22]) had no effect. This result led us to consider the possibility that, although PQS exhibits an inhibitory activity, its principal physiological role may be to elicit an efficient adaptive response.

### PQS reduces the intracellular level of reactive oxygen species

**Figure 5:**
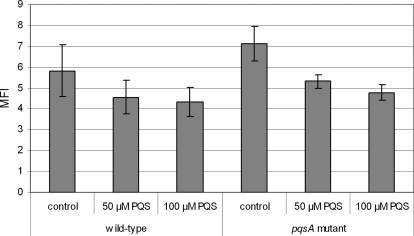
PQS reduces the intracellular ROI levels. The median of fluorescence intensity (MFI) of 50,000 log phase grown cells treated with 20 µM H_2_DCFDA is given. PQS was added to log phase grown PAO1 wild-type and *pqsA* mutant cultures at concentrations of 0, 50 and 100 µM PQS, respectively followed by an 1 h incubation period prior to FACS analysis. In contrast to PQS addition, the addition of at least equimolar concentrations of the biologically less active PQS precursor heptyl-4-hydroxy-quinoline (HHQ) or the iron chelator dipyridyl did not show a clear reduction of the ROI levels (data not shown). The assays were performed three times with independent cultures, the addition of PQS reduced the MFI significantly as determined by t-test (p<0.05).

Since PQS obviously sensitized the bacteria towards oxidative stress, albeit it was previously shown to induce the transcription of oxidative stress response genes [21], we decided to monitor the intracellular levels of reactive oxygen species (ROS) in mid-exponential phase grown cultures by employing the redox dye dihydro-dichloro-fluorescein diacetate (H_2_DCFDA). As shown in Figure 5, exogenous addition of PQS reduced ROS levels in both wild-type and *pqsA* mutant cells in mid-log phase cultures in a dose-dependent manner. The anti-oxidative effect of PQS was even observed when PQS was added immediately prior to FACS analysis (data not shown). In contrast to PQS, the addition of at least equimolar concentrations of HHQ or the iron chelator dipyridyl did not show a clear reduction of the ROS levels (data not shown).

### PQS is a primary anti-oxidant

**Figure 6:**
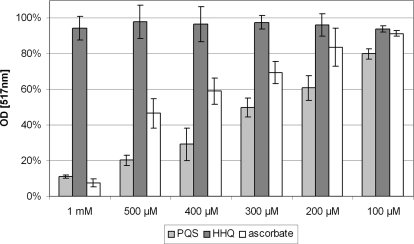
PQS is a primary anti-oxidant. The absorbance changes of the stable 2,2-diphenyl-2-picrylhydrazyl (DPPH) radical was measured in three independent experiments at the OD of 517 nm in the presence of PQS, HHQ and ascorbate. Error bars mark the standard deviation of three independent experiments. The decolorization is stoichiometric with respect to the number of electrons captured.

**Figure 7:**
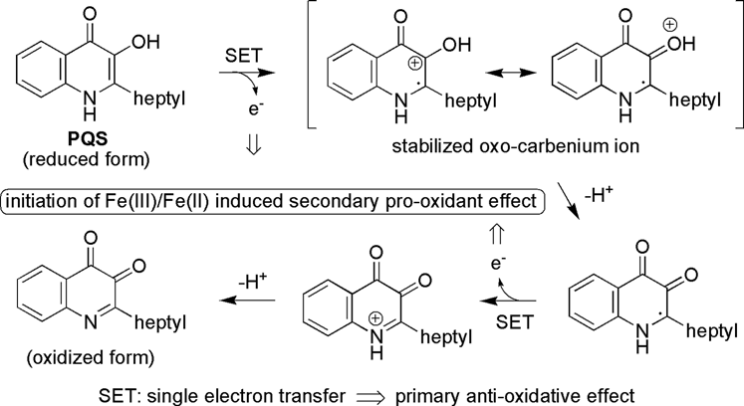
Proposed mechanism of the primary anti-oxidant effect and the secondary pro-oxidant effect of PQS.

To determine whether PQS is a primary anti-oxidant, we measured the absorbance changes of the stable 2,2-diphenyl-2-picrylhydrazyl (DPPH) radical in the presence of PQS. HHQ and ascorbate served as controls. As depicted in Figure 6, PQS is an anti-oxidant that exhibited an anti-oxidant activity comparable to that of ascorbate, whereas the HHQ molecule did not show any antioxidant activity. This observation is explicable by the much stronger electron-donating potential of PQS as compared to HHQ. From a structural perspective, this is due to the bisoxygenated aromatic system of PQS, which may readily be oxidized to the corresponding ortho-quinoid system. On the contrary, this process is much less pronounced for HHQ, which lacks the 3-OH group and may therefore not form directly a quinoid system (Figure 7).

### PQS is a pro-oxidant

**Figure 8:**
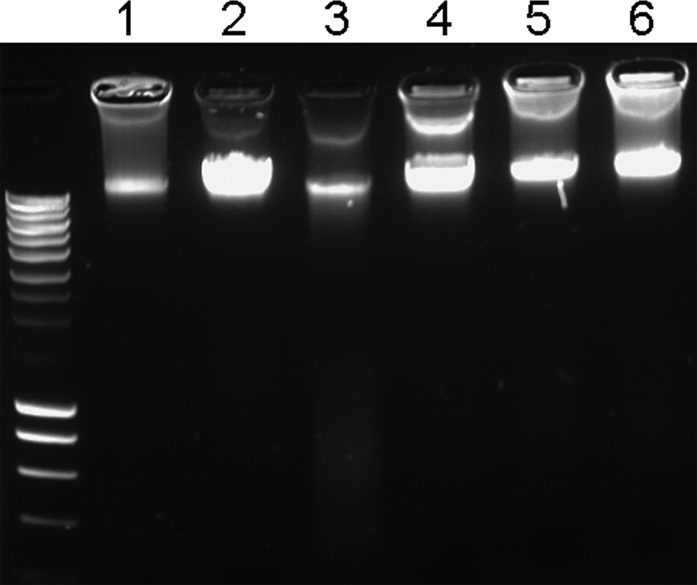
PQS exhibits pro-oxidants activities. The Fe2^+^ mediated DNA fragmentation process is strongly enhanced by the addition of PQS or vitamin C but not by the addition of the biological inactive precursor substance HHQ. Lane 1: chromosomal *P. aeruginosa* DNA under the addition of vitamin C and Fe2^+^, lane 2: DNA+vitamin C, lane 3: DNA , Fe2^+^ and PQS, lane 4: DNA and PQS, lane 5: DNA, Fe2^+^ and HHQ, lane 6: DNA and HHQ. The DNA was pre-incubated with PQS, HHQ or vitamin C, respectively, at room temperature for 45 min before exposure of the probes to 2.5 mM Fe2^+^ for 15 min and separation on an agarose gel.

**Figure 9:**
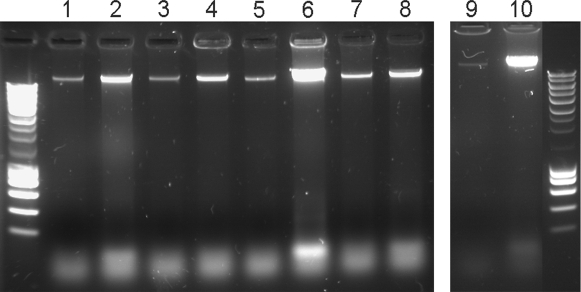
PQS dependent release of extracellular DNA. PAO1 wild-type, the *pqsA*, the *pqsH* and the *pqsL* mutant were grown in LB medium. Supernatant samples were collected after 8 h (lane 1, 3, 5, 7, 9) and 36 h (lane 2, 4, 6, 8, 10), respectively. Increasing amounts of fragmented DNA were found in the supernatant of the PAO1 wild-type cultures under prolonged incubation (lanes 1, 2). DNA release and fragmentation was delayed in the PAO1 *pqsA* and *pqsH* mutants (lane 3, 4 and lane 7, 8). The DNA release and fragmentation pattern was most pronounced in the *pqsL* mutant (lanes 5, 6) and the complemented *pqsA* mutant (*pqsA-E* operon on plasmid pLG10) (lanes 9, 10).

Antioxidants, such as vitamin C may also initiate pro-oxidative processes. For example, while the primary effect of vitamin C is an antioxidant activity by reduction of metal ions, this may lead to the generation of free radicals through the Fenton reaction, which themselves cause oxidative stress. Thus, molecules can act as either antioxidants, or pro-oxidants, depending on the specific set of conditions. Some of the conditions that are important include the concentration of the chemical and if oxygen or transition metals are present. Since PQS was shown to exhibit an antioxidant activity, but at the same time enhanced the susceptibility of the bacteria against external stresses, we wondered whether PQS also exhibits a pro-oxidant activity. We therefore tested the potential of PQS to enhance oxidative DNA damage. As depicted in Figure 8 the addition of PQS or ascorbate but not HHQ significantly enhanced DNA fragmentation in a cell free assay under the presence of iron ions. To demonstrate that this PQS induced oxidative DNA damage also plays a role *in vivo*, we monitored DNA release and fragmentation in growing *P. aeruginosa* cultures. We found that DNA was released to a significantly lower extent in the *pqsA* and a *pqsH* mutant as compared to the wild-type and the *pqsL* mutant cultures grown to stationary phase. Expression of the PQS biosynthetic operon *in trans* restored DNA release in the *pqsA* mutant (Figure 9).

Accordingly, PQS has a very similar effect as vitamin C, i.e. a primary antioxidant effect by its strong reducing potential forming a quinoid system, and an indirect second pro-oxidative effect, by initiation of radical mediated processes, presumably in a similar fashion as vitamin C by Fe(III)/Fe(II) mediated radical initiated oxidative processes (Figure 7). However, it is expected that this tendency may be even more pronounced for PQS due its strong tendency to coordinate Fe(III). This renders the further evaluation and study of this interesting system a potentially rewarding research goal.

### PQS is involved in resistance to UV irradiation

**Figure 10:**
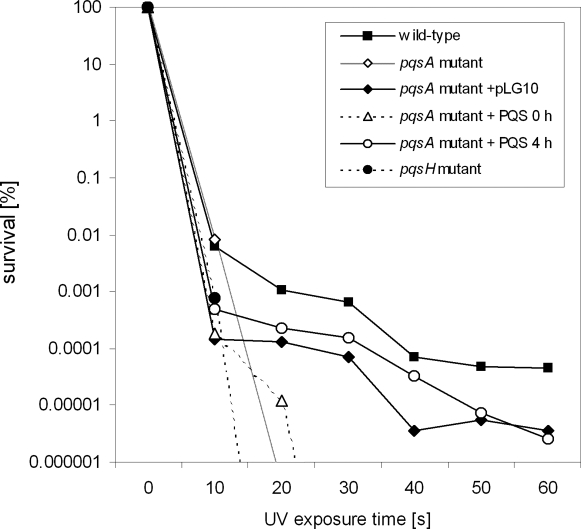
Killing curves upon exposure to UV irradiation. Wild-type PAO1, the *pqsA* mutant, the *pqsH* mutant and the *pqsA* mutant complemented with pLG10 were grown overnight in LB, and diluted in DeMoss medium [40]. The cells were UV irradiated while shaking, and aliquots were removed at 10s intervals. The exogenous addition of 100 µM PQS to the *pqsA* mutant cultures 4 h before exposure to UV radiation restored UV tolerance, whereas PQS addition immediately before the UV stress did not show any effect. Serial dilutions were plated on LB agar to determine the viable cell count. The UV killing assays were performed three times with independent cultures, and the outcome of one representative experiment is shown.

Since we could show in this study that PQS can induce oxidative DNA damage we wondered whether this DNA damage may trigger DNA repair and thus induce UV-tolerance. Therefore we applied UV-stress and monitored bacterial survival (Figure 10). Wild-type cells showed killing patterns, characterized by roughly 7 log units of killing over 60 s of UV irradiation. The *pqsH* and the *pqsA* mutant were hypersensitive to UV and were killed almost completely within 20–30 s of irradiation. The exogenous addition of 100 µM PQS and the expression of the *pqsA-E* operon *in trans* partially restored UV tolerance in the *pqsA* mutant.

## Discussion

Cellular autolytic systems are well known to participate in a drug-induced lyses of gram-positive bacteria [23]–[25] and mutants that lack these systems have been shown to become antibiotic tolerant [26]. Autolysis might be viewed as a maladaptive disbalance caused by the inhibition of cell growth, however it has also been speculated that autolysis represents a programmed response to stressful conditions [27]–[29]. Programmed cell death is defined as an active process that results in cell suicide and is recognized as an essential mechanism in multicellular organisms where it is required for eliminating damaged or potentially harmful cells, and may have evolved to aid the development of structured populations for the benefit of the population as a whole [29],[30]. In this study we provide evidence that a participation of a self-induced cell death in the drug-induced disintegration applies also to the gram-negative organism *P. aeruginosa*. We demonstrate that PQS mediates an endogenous stress that predisposes bacteria to developing exogenous stresses, compliant with the previous finding that overproduction of PQS leads to a highly autolytic phenotype that can be reversed by mutations in the PQS biosynthetic genes [19]. Most importantly, simultaneously, PQS induces the entry of undamaged bacteria into a less metabolically active, less susceptible state and is shown to be a potent antioxidant capable to reduce the intrabacterial ROS levels.

One of the most challenging adaptive responses in bacteria is to provide an oxidative stress response that allows the bacteria to optimally cope with reactive oxygen species (ROS) that are unavoidably generated during aerobic metabolism and to respond rapidly to exogenous ROS. Polymorphonuclear cells are the major effector cells responsible for the clearance of *P. aeruginosa* from the site of infection and an essential bacterial defence strategy of *P. aeruginosa* is the ability to withstand the oxidative stress that is induced during phagocytosis, when the bacteria are confronted with very high levels of reactive oxygen intermediates from the respiratory burst of human phagocytes.

The anti-oxidant activity of PQS is very similar to that of vitamin C which has previously been shown to exhibit both anti-oxidant as well as pro-oxidant activities [31]–[33]. The latter is due to the fact that ascorbate can reduce metal ions which leads to the generation of free radicals through the Fenton reaction. Addition of vitamin C to purified DNA in the presence of redox active metal ions has been shown to result in single-strand brakes and base modifications [34]–[36]. This is thought to be due to binding of the metal ion to the DNA and resultant site-specific hydroxyl radical production and oxidative damage [34]. In the absence of added metal ions, however, vitamin C inhibits the formation of base modifications in purified DNA exposed to peroxynitrite or UV light [35],[37],[38]. In this study we have clearly demonstrated that not only ascorbate but also PQS strongly enhances DNA fragmentation in the presence of oxygen and metal ions *in vitro*, and monitoring of DNA release in stationary *P. aeruginosa* cultures *in vivo* revealed that PQS significantly enhanced DNA release and fragmentation. However, most importantly, at the same time PQS provides the bacteria with an increased fitness under UV radiation. It seems that damaged DNA may serve as a sensor for stressful conditions and thus may trigger DNA repair systems essential for the development of UV tolerance.

We suggest that the identification of the interbacterial *Pseudomonas* signaling molecule, PQS, as a factor that exhibits both pro- and anti-oxidant activities points to PQS as a cellular “trainer” whose role is to mediate selection of the fittest through a “make or break” mechanism. PQS seems to issue decisions on life and death in *P. aeruginosa* populations, and thereby might be essential to shape the population structure, contribute to multi-cellular development processes in bacterial biofilms, and thus help to maintain discrete, ordered spatial structures. It is interesting to speculate that fragmentation of cellular populations may be a widespread stratagem for clonal survival under stressful conditions; it further raises the prospect that the factors involved in this process might ultimately be exploited as novel targets for anti-bacterial interventions that may even be effective in the treatment of chronic biofilm infections.

## Materials and Methods

### Bacterial strains and culture conditions

PAO1 and isogenic transposon mutants with an insertion within the *pqsA, pqsH* and *pqsL* genes were obtained from the Washington Genome Center. The *P. aeruginosa* strains were routinely cultured at 37°C on Luria-Bertani (LB) agar or LB broth. To restore 4-quinolone production in the *pqsA* mutant the bacteria were transformed with the plasmid pLG10 (kindly provided by Colin Manoil) harboring the *pqsA-E* operon. To monitor a decrease in bioluminescence after exposure to the antibiotic ciprofloxacin, a *luxCDABE* operon was introduced in trans into the *P. aeruginosa* strains. Plasmid containing bacteria were cultured in LB under the addition of 500 µg/ml carbenicillin.

### Killing curves

To monitor viability after antibiotic exposure, the *P. aeruginosa* PAO1 wild-type and the respective mutants were grown in LB broth to the same OD_600_ in mid logarithmic phase before the bacteriocidal agents were added. Ciprofloxacin was used at a concentration of 5 µg/ml, imipenem and gentamicin at a concentration of 7.5 µg/ml. OD_600_ values were obtained at the indicated time intervals and CFU counts were determined by plating appropriate dilutions of the bacterial suspension on LB agar plates.

### Determination of extracellular DNA

For the determination of the release of DNA by *P. aeruginosa* cultures, the bacteria were grown in LB medium for up to 36 h, the cultures were centrifuged at 13.000 rpm for 10 min and the supernatant was mixed with loading buffer. The probes were subjected to agarose gel electrophoresis using a 1% agarose gel stained with GelStar Nucleic Acid Gel Stain (Lonza, USA) and photographed over UV-light.

### Growth curves

To monitor the dependency of bacterial growth on PQS we exposed *pqsA* PAO1 mutant cultures to different concentrations of synthesized PQS [21]. Synthesized 2-heptyl-4-hydroxy-quinoline (HHQ) and 2,2′-dipyridyl served as controls. As opposed to the PAO1 wild-type, the *pqsA* mutant did not form clumps nor did the mutant produce pyocyanin so that the optical density could be monitored for prolonged incubation periods. Bacteria were cultured in LB medium in flasks shaken at 180 rmp.

### Flow cytometry

Fluorescence-activated cell sorter (FACS) cytometry analysis was performed using the H_2_O_2_-activated green fluorescent dye redox dye dihydro-dichloro-fluorescein diacetate (H_2_DCFDA, Molecular Probes). This dye is readily taken up by cells, cleaved by cellular esterases to produce non-cell permeant H_2_DCF, which can be activated by ROS giving green fluorescent DCF as an indication of intracellular levels of oxidative stress.

The bacteria were cultured overnight in LB, diluted to an OD_600_ of 0.05 and grown to mid-exponential phase with and without the addition of various PQS concentrations. The 4-quinolone 2-heptyl-4-hydroxy-quinoline (HHQ) at equimolar concentrations and the iron chelator dipyridyl served as controls. Cells suspensions were adjusted to an OD_600_ of 0.5, pelleted by centrifugation, and resuspended in phosphate buffered saline (PBS) containing 20 µM H_2_DCFDA. The suspension was incubated for 4 h, diluted 1∶100 in PBS and the fluorescence levels of 50,000 cells were recorded using a FACScalibur cytometer (BD Biosciences). Summit software (Dako Colorado) was used for data analysis.

### Antioxidant activity

To measure the radical-scavenging activity of PQS we utilized the stable 2,2-diphenyl-2-picrylhydrazyl (DPPH) radical. The odd electron in the DPPH free radical gives a strong absorption maximum at 517 nm and is purple in color. The color turns from purple to yellow as the molar adsorptivity of the DPPH radical at 517 nm reduces from 9660 to 1640 when the odd electron of DPPH radical becomes paired with a hydrogen from a free radical scavenging antioxidant to form the reduced DPPH-H. 1 mg/ml DPPH in methanol/water (90/10; vol/vol) was incubated for 15 min at room temperature and the absorbance changes were measured at 517 nm. The 4-quinolone HHQ and ascorbate served as controls.

### H_2_O_2_ sensitivity assay

The H_2_O_2_ sensitivity disk assay was adapted from Hassett and colleagues [39]. Briefly, PAO1 strains were grown at 37°C in BHI medium. One hundred micoliters of the bacterial culture was suspended in 3 ml of LB soft agar at 40°C (0.6% (w/v) agar) mixed and poured on LB agar plates. Sterile filter paper disks were placed on the soft solid agar and the disks were spotted with 8 µl of 30% H_2_O_2_. Plates were incubated for 24 h and the diameter of the zone of growth inhibition was measured. All experiments were performed at least in triplicates.

### UV irradiation sensitivity assay

200 µl of overnight LB-grown bacteria were diluted 1∶10 in LB medium, grown for another 4 h, with or without the addition of 100 µM PQS, and finally diluted 1∶10 in DeMoss medium [40]. The bacterial suspension was transferred into a glass petri dish at room temperature. Irradiation was performed with a UV lamp (LTF Labortechnik, VL-208G) placed 11 cm above the cells. Samples were removed at 0, 10, 20, 30, 40, 50 and 60 sec of UV irradiation, serially diluted and spotted on LB agar to determine the CFU counts after overnight incubation.

## Supporting Information

Figure S1 Growth behavior of PAO1 wild-type and mutant strains. Growth of the PAO1 wild-type, the *pqsA*, *pqsH*, *pqsL* mutants and the complemented *pqsA* mutant were comparable.
(0.08 MB TIF)

Figure S2 Antibiotic tolerance in the absence of PQS production. Killing curves of log phase grown *P. aeruginosa* PAO1 cultures treated with 5 µg/ml ciprofloxacin (A). Killing was significantly delayed in the *pqsA* mutant compared to the wild-type as determined by OD_600_ determinations and CFU counts after 3 h of exposure and could be reversed by the introduction of the *pqsA-E* operon *in trans*. Killing curves of bacterial cultures treated with 7.5 µg/ml gentamicin (B), and 7.5 µg/ml imipenem (C) were also recorded. Error bars mark the standard deviation of three independent experiments. CFU counts of the PAO1 wild-type and the *pqsA* mutant were significantly different (p<0.05, as determined by t-test).
(0.24 MB TIF)
