# Supplementary figures and images for: The Pseudomonas Quinolone Signal (PQS) Balances Life and Death in Pseudomonas aeruginosa Populations
Source: PLoS Pathog. 2008 Sep 26;4(9):e1000166. doi: 10.1371/journal.ppat.1000166 (PMC2533401; doi:10.1371/journal.ppat.1000166)

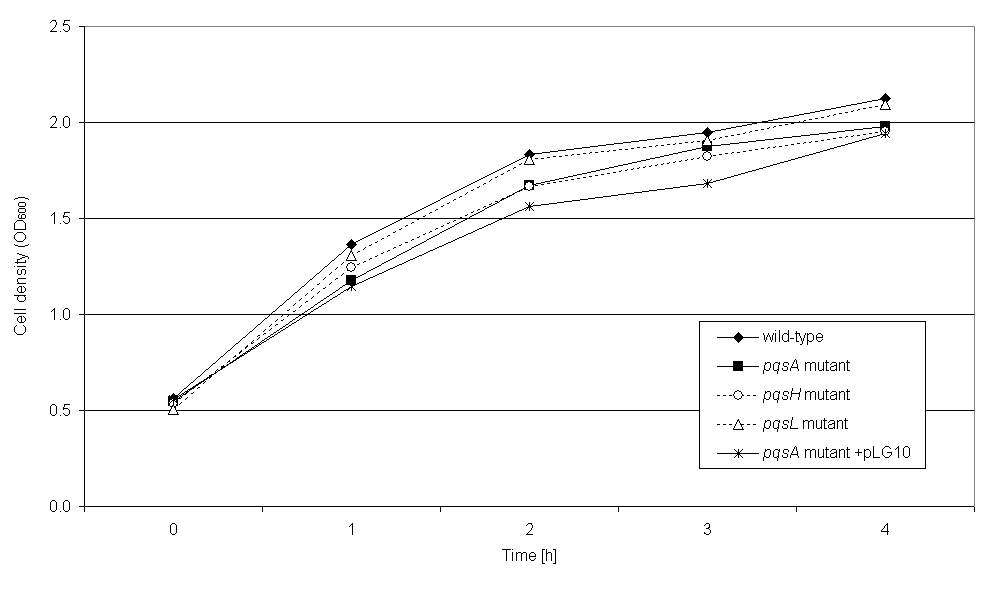

Supplement: Figure S1 — Growth behavior of PAO1 wild-type and mutant strains. Growth of the PAO1 wild-type, the pqsA, pqsH, pqsL mutants and the complemented pqsA mutant were comparable. (0.08 MB TIF) [file ppat.1000166.s001.tif]

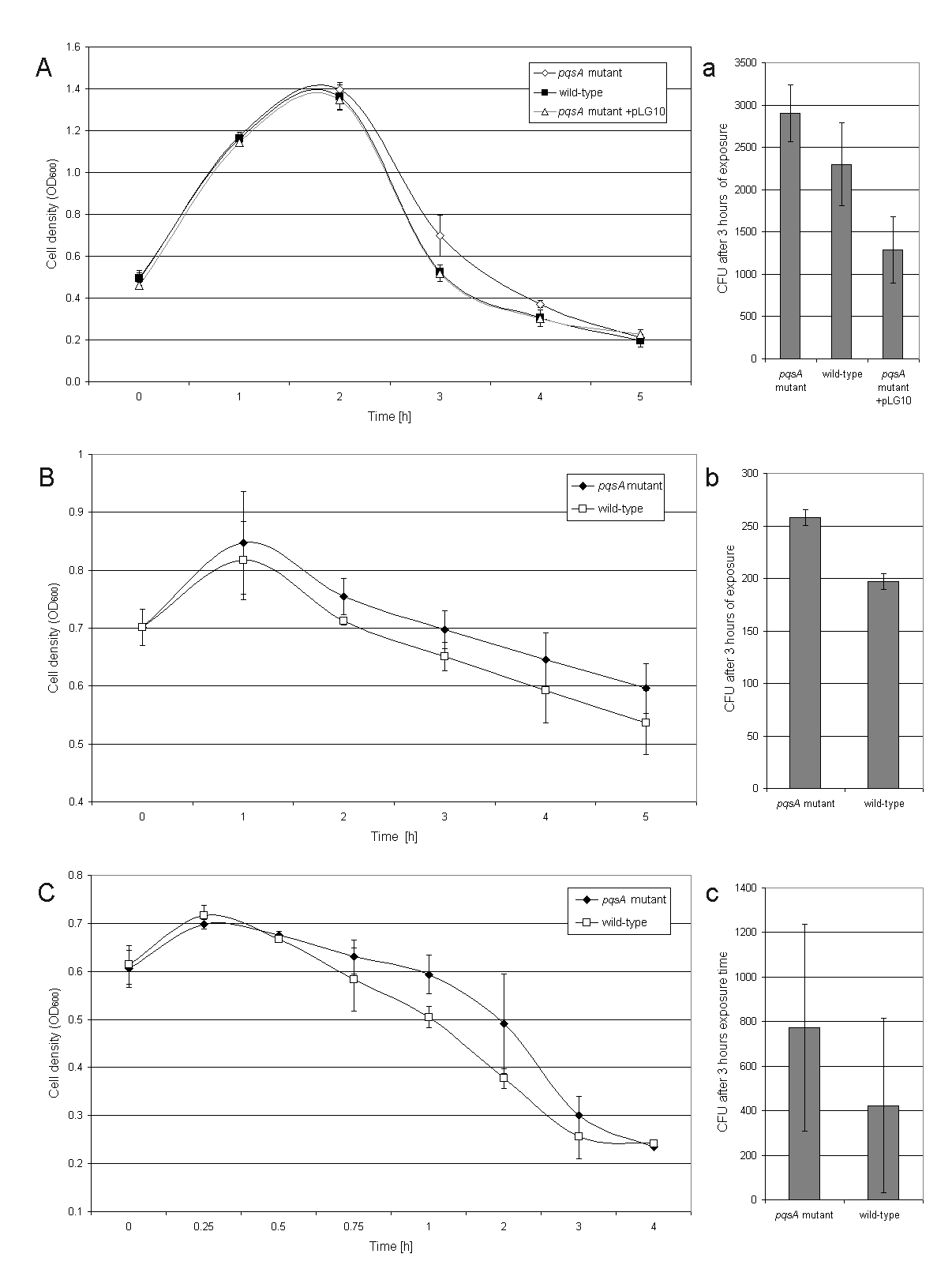

Supplement: Figure S2 — Antibiotic tolerance in the absence of PQS production. Killing curves of log phase grown P. aeruginosa PAO1 cultures treated with 5 µg/ml ciprofloxacin (A). Killing was significantly delayed in the pqsA mutant compared to the wild-type as determined by OD600 determinations and CFU counts after 3 h of exposure and could be reversed by the introduction of the pqsA-E operon in trans. Killing curves of bacterial cultures treated with 7.5 µg/ml gentamicin (B), and 7.5 µg/ml imipenem (C) were also recorded. Error bars mark the standard deviation of three independent experiments. CFU counts of the PAO1 wild-type and the pqsA mutant were significantly different (p<0.05, as determined by t-test). (0.24 MB TIF) [file ppat.1000166.s002.tif]
